# Sucrose Metabolism and Transport in Grapevines, with Emphasis on Berries and Leaves, and Insights Gained from a Cross-Species Comparison

**DOI:** 10.3390/ijms22157794

**Published:** 2021-07-21

**Authors:** Robert P. Walker, Claudio Bonghi, Serena Varotto, Alberto Battistelli, Crista A. Burbidge, Simone D. Castellarin, Zhi-Hui Chen, Philippe Darriet, Stefano Moscatello, Markus Rienth, Crystal Sweetman, Franco Famiani

**Affiliations:** 1 Independent Researcher, Bolton BL2 3BG, UK; 2 Department of Agronomy, Food, Natural Resources, Animals and Environment, University of Padova Agripolis, 35020 Legnaro, Italy; serena.varotto@unipd.it; 3 Istituto di Ricerca sugli Ecosistemi Terrestri, Consiglio Nazionale delle Ricerche, 05010 Porano, Italy; alberto.battistelli@cnr.it (A.B.); stefano.moscatello@cnr.it (S.M.); 4 Agriculture and Food (CSIRO), Glen Osmond, SA 5064, Australia; Crista.Burbidge@csiro.au; 5 Wine Research Centre, Faculty of Land and Food Systems, University of British Columbia, Vancouver, BC V6T 0Z4, Canada; sdcastel@mail.ubc.ca; 6 College of Life Science, University of Dundee, Dundee DD1 5EH, UK; z.y.chen@dundee.ac.uk; 7 Cenologie, Institut des Sciences de la Vigne et du Vin (ISVV), 33140 Villenave d’Ornon, France; philippe.darriet@u-bordeaux.fr; 8 Changins College for Viticulture and Oenology, University of Sciences and Art Western Switzerland, 1260 Nyon, Switzerland; markus.rienth@changins.ch; 9 College of Science & Engineering, Flinders University, GPO Box 5100, Adelaide, SA 5001, Australia; crystal.sweetman@flinders.edu.au; 10 Dipartimento di Scienze Agrarie, Alimentari e Ambientali, Università degli Studi di Perugia, 06121 Perugia, Italy

**Keywords:** fruits, grapevines, invertase, leaves, metabolism, osmoregulation, sucrose, transcripts, transport, *Vitis vinifera*

## Abstract

In grapevines, as in other plants, sucrose and its constituents glucose and fructose are fundamentally important and carry out a multitude of roles. The aims of this review are three-fold. First, to provide a summary of the metabolism and transport of sucrose in grapevines, together with new insights and interpretations. Second, to stress the importance of considering the compartmentation of metabolism. Third, to outline the key role of acid invertase in osmoregulation associated with sucrose metabolism and transport in plants.

## 1. Introduction

Sucrose and its constituents, glucose and fructose, account for the bulk of the non-structural carbohydrate content of many ripe fruits, including grapes. These sugars have a number of functions, which include: providing a metabolic substrate, acting as a carbon store, osmoregulation, transport, signalling and, in their wild ancestors, enticing animals to disperse the fruits/seeds [1]. In this article, the transport and metabolism of sucrose in grapevines is reviewed. Sugar signalling in grapevines and other plants has been reviewed [2,3,4], as has the effect of hormones on sink strength in grapevines [4], and are not considered in this review. Other species are considered because these can provide useful insights. The importance of considering the compartmentation of metabolism is stressed. Further, the key role of acid invertase in osmoregulation associated with the transport and metabolism of sucrose is examined.

## 2. Grapevine Structure and Metabolism

Although studies using whole organs/tissues have provided the backbone of our knowledge regarding grapevine metabolism, they often lead to questions that can only be answered if structure and compartmentation are considered [5,6,7,8].

### 2.1. Compartmentation between Organs

Grapevines consist of a number of organs (such as the leaf, root, stem and berry), and because sucrose is transported between them, their sugar metabolism is linked. The bulk of the sugar content of grapevines is produced by leaf photosynthesis and is exported from leaves as sucrose in the phloem [9,10,11]. The destination of the exported sucrose depends on factors such as leaf position and the phenological stage of the grapevine. For example, after the fruit set, there is apical movement of assimilates from 4–5 leaves below the shoot tip. At veraison, basal movement predominates and increases steadily with the developing berries, and during ripening, there is practically no apical movement of assimilates [12]. Grapevines are temperate deciduous woody vines that store starch in their roots and stems. This is converted to sucrose, which is then used to support the growth of developing shoots and other sinks when growth resumes in the spring [11,13,14,15].

### 2.2. Compartmentation between Tissues/Cell Types

The organs of grapevines are composed of a number of tissues, and these contain different cell types. For example, vascular tissues contain xylem element, sieve elements and their associated companion cells together with a variety of different parenchyma cells. The metabolism of these can differ considerably, and to more fully understand their metabolism, studies that investigate individual tissues and cell types are required [5,7,8,16,17,18,19]. Many enzymes and sugar transporters are localised in certain tissues/cells, and it is important to know these locations in order to begin to understand their functions [5,6,7,11]. Nevertheless, even when these locations are known, a further greater difficulty in determining their role is often encountered, that is, the lack of understanding of the metabolism/functioning of the tissues/cell types in question. For example, although acid invertase (AI) and phosphoenolpyruvate carboxykinase (PEPCK) proteins were localised in various cell types in grape berries, their functions remained largely enigmatic [5,6,7]. To begin to understand their functions have then required many years of tortuous work [6,20,21,22].

### 2.3. Compartmentation within Cells and between Apoplast and Symplast

Metabolism is compartmentalised within cells and between the apoplast and symplast. The contents of sugars and the enzymes that metabolise them differ between cell compartments (e.g., vacuole, plastid and cytosol) [8,18,23,24,25,26,27]. The membranes that enclose these compartments require transporters to allow rapid passage of sugars across them, and the transporters present depend on the membrane in question as well as other factors such as the species, cell type and stage of development [11,26]. When large fluxes of sugars occur (e.g., apoplastic phloem unloading in sinks and apoplastic loading in leaves), it is necessary for the membrane to have a sufficient quantity of aquaporin water channels; because these enable a rapid osmotically driven bulk flow of water [28].

### 2.4. Compartmentation of Sugars in Leaf Mesophyll Cells and Pericarp Parenchyma Cells

In mesophyll cells of mature leaves, the vacuole usually occupies a lower proportion of cell volume (68–85%) [24] than in ripening grape pericarp (about 99%) [29]. This alters the volume occupied by the cytoplasm, which in ripe grape berries must be around 1%, whereas, in leaf mesophyll cells, it occupies 15–32% of cell volume [24]. Hence in leaves, a greater proportion of their sugar content is likely to be located in the cytoplasm than in the pericarp. In mesophyll cells, a large proportion of glucose and fructose is contained in the vacuole [18,24,30], and their concentrations in the vacuole are often much higher than in the cytoplasm [24,30]. By contrast, the bulk of the mesophyll sucrose content is often located in the cytoplasm [18], and its concentration can be 2–20-fold higher than in the vacuole [24]. However, the proportion of sucrose contained in the vacuole is dependent on a number of factors, and environmental conditions that limit sink demand can lead to a substantial increase [31]. Similarly, in grape leaves, sucrose content can increase during the day under certain conditions [32], and it is possible that the sucrose in excess of that required for export is stored in the vacuole [33].

### 2.5. Distribution of Sugars between Symplast and Apoplast

The concentration of sugars in the bulk apoplast of source leaves and ripening fruit flesh is very different. Sucrose concentration in the bulk leaf apoplast is low and often between 0.1–6 mM [24,34]. If sugars are present in the leaf apoplast, they are carried by the transpiration stream away from the phloem [34,35]. Hence, sucrose destined for export is assumed to be transported to the phloem in the symplast [36].

In grape pericarp, there is a large increase in the apoplastic concentrations of glucose and fructose around the onset of ripening, and these then remain high. These hexoses are the predominant apoplastic osmoticum, with a total concentration of around 800 mM, and are important in lowering parenchyma cell turgor that is necessary for fruit softening [37,38]. Similarly, in ripe sweet cherry flesh (and in the ripe flesh of sour cherry, European plum, tomato and a range of soft fruits), the most likely explanation for the low turgor pressure of the parenchyma cells is a build-up of apoplastic solutes [39,40]. According to Schuman et al. [40], the apoplastic volume is only around 10% of cell volume in cherry flesh. This compares with 3–8% for tomato flesh [41], and in kiwifruit flesh, the value increased from 7.5–20% during development [42]. Thus, the bulk of sugar content is within the cell. The very low content of sucrose in the flesh of cherry (Table 1; [1]) indicates that a large proportion of the apoplastic sugars consists of glucose and fructose. Ripe cherries, grapes and cultivated tomatoes (*S. lycopersicon*), all accumulate large amounts of glucose and fructose but little sucrose (Table 1).

In contrast, in ripening fruits that accumulate quite large amounts of sucrose, there is evidence that the ratio of sucrose:(glucose + fructose) (S:(G+F)) in the apoplast is higher. Thus, a comparison of S:(G+F) of the symplast and apoplast of apple [56], grape [37], kiwifruit [42], melon [57], peach [58], tomato (*S. lycopersicon*) [41,59] and strawberry [60] indicates that for a given fruit, S:(G+F) of the symplast is positively correlated with that of the apoplast. Indeed, in sugar cane internodes, there is a striking correlation between S:(G+F) of the symplast and apoplast [61]. Thus, as the internode ages, the amount of hexose in both the apoplast and symplast declines greatly [61]. Further evidence for this correlation is provided by asymmetrically labelled sucrose studies in tomatoes. These showed that only a small proportion of sucrose was hydrolysed in the apoplast if the fruit mainly stored sucrose, and a large proportion was if it mainly stored hexoses [41,62,63].

## 3. Sucrose Metabolism Enzymes and Transporters

**Figure 1:**
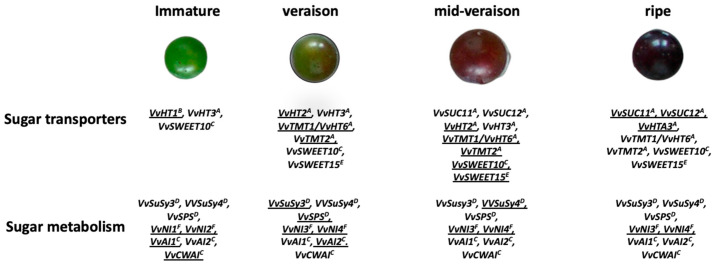
Schematic representation of transcript abundance of sugar metabolism enzymes and sugar transporters in the pericarp of *Vitis vinifera* during development. Underlined genes are the ones whose transcripts are most abundant at the given stage of development. Letters correspond to the following references: (A) [74]; (B) [75]; (C) [76]; (D) [77]; (E) [78]; (F) [79]. Additional gene information is given in Supplementary Table S1. Abbreviations: AI = acid invertase; CWAI = cell wall acid invertase; HT = hexoses transporters; NI = neutral invertase; SWEET = sucrose will eventually be exported transporter; TMT = tonoplast monosaccharide transporter; SuSy = sucrose synthase; SPS = sucrose phosphate synthase. Ripening stages (veraison, mid-veraison and ripe) are those defined by Coombe [80] and Fasoli et al. [81].

In grapevines, much attention has been focussed on certain key regulatory enzymes. Nevertheless, transcriptomics and proteomics have provided information regarding the occurrence/abundance of both much and little-studied enzymes as well as sugar transporters (Figure 1; Supplementary Table S1; [64,65,66,67,68,69,70,71,72]). Results of studies in which the abundance of various enzymes involved in sucrose metabolism was altered by transgenic manipulation in tomatoes are summarised by Beckles et al. [73].

Regulatory enzymes are subject to fine control (changes in enzyme/transporter activity not brought about by changes in the abundance of the protein); others termed housekeeping enzymes are not [82]. Fine control is complex; because there are numerous interactions between a plethora of mechanisms. Post-translational modifications such as phosphorylation, which can alter substrate affinities, interactions with metabolite effectors and regulatory proteins, are often modulated by pH and other factors. Recently, also glycation has been reported as a possible way for enzyme activity modulation [83]. PEPCK, sucrose synthase (SuSy), sucrose phosphate synthase (SPS) and the invertases are all known to be modulated by many of these factors [3,84,85,86,87,88]. The situation is made more complex because of the occurrence of metabolons and substrate channelling, that is, a temporary structural-functional complex formed between consecutive enzymes, which permits the intermediate product from one enzyme to be channelled into the active site of the next one of the considered pathways. An example is the association of glycolytic enzymes with each other by protein–protein interactions and their association with the mitochondria when respiratory demand is high [89]. Thus, measurements of enzyme activity *in vitro* often do not reflect activity *in vivo*. Few studies of enzyme regulation by fine control have been performed in grapevines (e.g., [90,91]).

### 3.1. Entry of Sucrose into Metabolism

Sucrose enters metabolism via invertase (sucrose + water → glucose + fructose) or SuSy (sucrose + UDP ↔ UDP-glucose + fructose) [4,92]. There are two groups of invertases, the neutral and acid invertases [86]. In addition to having a physiological role, both the invertases and SuSy can function in sugar sensing and signalling [3,93]. The activities of SuSy and the invertases in grape pericarp and the flesh of some other fruits before and during ripening are shown in Table 2.

#### 3.1.1. Sucrose Synthase

SuSy is encoded by a small gene family, and, although the enzyme is often located in the cytosol, it can be present in other locations, such as in association with the plasma membrane [4,93]. The *V. vinifera* genome contains five SuSy genes [104]. In plants, SuSy genes can be divided into three clades [93,104]. In grape, *VvSS4* belongs to clade I, *VvSS2* and *VvSS3* to clade II and *VvSS1* and *VvSS5* to clade III [104]. The abundance of transcripts arising from each SuSy gene is dependent on tissue and stage of development. *VvSS1*, *VvSS2* or *VvSS5* transcripts are at low abundance in all tissues, whilst *VvSS3* and *VvSS4 t*ranscripts are abundant in certain tissues, such as the pericarp or seed [104]. SuSy has diverse functions, which include providing substrate for biosynthesis, which is particularly important in the vasculature and developing fruits/seeds in which O_2_-supply is low [72,93].

#### 3.1.2. Neutral Invertase

Neutral invertase (NI) is encoded by a small gene family, which can be subdivided into the α (located in organelles) and β (located in the cytosol) neutral invertases. The *V. vinifera* genome contains four α and five β *NI* genes, and transcript abundance from each gene is tissue-dependent [79]. However, a recent study identified further 2 NI genes [4]. Cytosolic NI plays a key role in providing substrate for metabolism. NI is also likely important in osmoregulation in different cytoplasmic compartments [79,105,106,107,108].

#### 3.1.3. Acid Invertase

Acid invertase (AI) is encoded by a small gene family, which can be divided into two clades; one encodes vacuolar acid invertase (VAI) and the other cell wall acid invertase (CWAI) [86]. Two genes for vacuolar and four genes for CWAI have been detected in the *V. vinifera* genome [103,109]. However, a recent study identified 3 VAI genes and 5 CWAI genes [4]. Symplast:apoplast solute concentration plays a key role in determining cell turgor pressure; a high ratio increases turgor by drawing water into the cell by osmosis, whereas a low ratio does the reverse. AI hydolyses sucrose to glucose plus fructose, which doubles the osmotic effect [86]. This enzyme can be located in the cell wall or vacuole and can therefore contribute to increasing or decreasing turgor pressure. AI can be inactivated by binding to AI inhibitor protein; this is a highly regulated process that can rapidly allow activation/inactivation of the enzyme [110,111]. Therefore, a lack of correlation between the presence of the enzyme and the abundance of sucrose/glucose/fructose does not rule out a physiological role because the active enzyme might only be required under certain circumstances. AI is generally regarded as providing substrate for metabolism; however, in many situations, the glucose and fructose produced by AI are primarily used in osmoregulation.

### 3.2. Sugar Transporters

The molecular identity of the sugar transporters involved in some membrane transport processes was determined in some plant species; this allowed a number of these to be cloned from grapevine [4,11]. The availability of genome data and bioinformatics allowed sugar transporters to be organised into families. Sugar transporters can be divided into three families: sucrose uptake transporters (SUT/SUC: active sucrose/H+ symporters), hexose transporters (HT: active hexose/H+ symporters) and sucrose will eventually be exported transporters (SWEETs), a passive facilitator [2]. The *V. vinifera* genome contains 4 SUTs, 59 putative HTs and 16 putative SWEETs [2,4]. Sequence data do not reveal transport properties. Transporters have to be cloned, expressed in yeast, and then sugar uptake properties can be determined [11].

Uptake/release studies of radiolabelled sugars indicate that in many plant tissues, both active and passive transport processes are involved in sugar transport across membranes [41,112,113]. Linear passive uptake from the apoplast can potentially be brought about by either SWEETs or pinocytosis/endocytosis [76,114,115]. Studies of protoplasts and vacuoles isolated from grape pericarp indicate that both active and passive processes are involved [116].

The membrane in which the transporter is located (e.g., plasma membrane or tonoplast) can be determined using transient expression in amenable plant tissues [11,76]. Sugar transporter transcript abundance gives information as to potential transport capacity [11]. Quantitative proteomic studies of purified plasma membranes and vacuoles from grape can provide information regarding transporter protein abundance [27,117]. The activity of sugar transporter proteins is subject to regulation [118,119]; however, very little is known about this in grapevines [11]. Expression studies in amenable species can provide useful insights into transporter function [76,120]. In order to understand function further, it is necessary to determine in which cell types a transporter is present, and few such studies have been performed in grape [2,11,121].

#### 3.2.1. Sucrose Transporters

Transcript abundance of *VvSUC11* (Km sucrose 0.9 mM), *VvSUC12* (Km sucrose 1.4 mM) and *VvSUC27* (Km sucrose 8–10 mM) is dependent on tissue and stage of development [11,74]. In the pericarp, the abundance of both *VvSUC11* and *VvSUC12* transcripts increase at veraison, whereas those of *VvSUC 27* decrease [2,11]. *VvSUC11/12/27* transcripts are present in leaves [11,74], and functional analysis of *VvSUCs* in Arabidopsis pointed out the potential of these transporters to function in phloem loading [120]. A fourth distinct sucrose transporter sequence, *VvSUT2* is present in grapes; however, transcript abundance is low in most tissues [10].

#### 3.2.2. Hexose Transporters

Transcript for six HT gene homologues (*VvHT1-6*) are present in the pericarp; three are likely plasma membrane-located (*VvHT1, 4, 5*), and two appear to be tonoplast-located (*VvHT2* and *6*) [2,11,122]. VvHT1, 4, 5 are glucose transporters (*VvHT1* Km glucose 70 µM; *VvHT4* Km glucose 150 µM; *VvHT5* Km glucose 100 µM). *VvHT1* and 5, but not *VvHT4*, can also transport fructose. Transcripts for *VvHT1-3* and particularly *VvHT3* are the most abundant in the pericarp [2]. *VvHT1* is localised in the conducting elements of the phloem of young berries, and transcripts are abundant in berries before ripening and in developing leaves [121]. *VvHT6* transcripts are abundant at veraison [2]. Transcripts for *VvHT1*, 3, 5 increase as the leaves matures, as does a CWAI transcript, and HTs could play a role in the retrieval of apoplastic hexose [75].

#### 3.2.3. SWEET Transporters

Zhang et al. [76] characterised *VvSWEET10* in grape pericarp: transcript abundance increased at veraison, it was located in the plasma membrane, transported glucose and fructose, and transcripts were present in parenchyma and vasculature. Over-expression in tomato increased fruit sugar content. *VvSWEET10* is likely to play a role in glucose and fructose uptake by parenchyma cells during ripening [76]. This does not preclude a role for HTs; because in tomatoes, there is evidence that both SWEETs and HTs are involved [41,76,123,124]. Breia et al. [125] determined the transcript abundance of the different SWEET genes during pericarp development. Transcripts arising from some genes were not detected, and transcript abundance from those that were detected was dependent on the stage of development. *VvSWEET7* (highest abundance at the green stage; transports hexoses and sucrose) and *VvSWEET15* (highest abundance at the mature stage; transport activity not detected) were plasma membrane-located [125].

## 4. Pericarp Sugar Metabolism

The sugar contents of the grape pericarp and the flesh of some other fruits before and after ripening are shown in Table 1. Glucose and fructose account for the bulk of the sugar content of the pericarp of *V. vinifera* cultivars throughout development (Table 1; [126]). In some cultivars that are derived (at least in part) from other *Vitis* species, sucrose can account for about 20% of the soluble sugar content in the flesh and up to about 30% in the skin of the ripe pericarp (Table 1; [43,94,127]). Glucose and fructose contents depend on the stage of development, cultivar, cultural practices and growth conditions. Although glucose:fructose is often close to one, it can be much higher, especially at elevated temperatures; it also depends on the stage of development [128,129,130]. The reasons for these deviations from unity are not understood and are likely complex [1,131]. In all grape cultivars, as in many other fruits, the total content of soluble sugars is quite low before the start of ripening and then increases (Table 1; [11,43,94,130,132]). Thus, after the onset of ripening, a much lower proportion of imported sucrose is used in processes that transform sucrose into compounds other than glucose and fructose. Diurnal changes in sucrose content, of about 25%, have been found during ripening [133]. The starch content of both the flesh and skin is low throughout development (<0.5 mg g^-1^ FW; [77]). Transcript abundance of some enzymes and transporters during pericarp development are shown in Figure 1.

Imported sucrose provides the bulk of the substrate used by metabolism, and during the ripening of grape pericarp, about 9–14% of imported sucrose is catabolised to CO_2_ [134]). For a few days following veraison, the vacuolar store of malic acid can provide a large proportion of metabolic substrate, and a small proportion of malate can be converted to sugars [48,134,135]. PEPCK is a key enzyme used in catalysing the gluconeogenic flux from malate to sugars in grape pericarp [5,21,48,136,137,138,139].

### 4.1. Sucrose Synthase

SuSy is present throughout development, and the amounts of its enzymatic activity (about 0.8–5.9 µmol g-1 FW h^−1^) depend on the cultivar and stage of development [78,94]. Some studies have found that SuSy activity g^−1^ FW increases during ripening [43,92], whereas others did not [94]. *VvSS3* transcripts are far more abundant than those of other SuSy genes, and this high abundance starts around veraison [104]. *VvSS4* transcripts are much more abundant than those of *VvSS3* in developing seeds [104].

SuSy has an important role in sucrose breakdown in tissues in which O_2_-supply is low; because compared to invertase, SuSy conserves energy (it produces UDP-glucose as opposed to glucose) [93]. In grape berries and other fruits, O_2_-supply is limited, and the flesh is often hypoxic [140,141,142]. In starch accumulating fruits such as kiwifruit, and other plant tissues, SuSy plays an important role in providing substrate for starch synthesis that is related to this energy-conserving aspect [98,143]. Vascular tissues are often hypoxic, and in these, SuSy is likely to play a role in providing substrate for cellulose, callose and starch synthesis [144,145]. It is likely that SuSy functions likewise in grapevine vasculature.

Although either SPS or SuSy can potentially be used in sucrose synthesis, the predominant route in most tissues is via SPS [85]. Nevertheless, it appears that SuSy can make an important contribution in some tissues, including fruits and sugar beet tubers [146,147,148,149,150]. There is evidence that the kinetic properties of certain SuSy isoforms enhance their activity towards sucrose synthesis and that post-translation modifications can also contribute [123,150]. Synthesis of sucrose using the SuSy pathway requires less ATP than the SPS pathway. This is because fructose is the substrate for SuSy and fructose-6-phosphate is the substrate for SPS. Hawker [92] reported not dissimilar amounts of SuSy and SPS activity in grape pericarp during ripening. It is possible that SuSy functions in sucrose synthesis in grape pericarp; however, such a role requires more evidence.

### 4.2. Neutral Invertase

When an invertase activity assay is conducted at either acidic or alkaline pH, both NI and AI contribute to the activity: because the pH range of activity of both enzymes is broad [97,151]. In grape pericarp, which contains large amounts of AI, it is probably impossible to reliably determine the activity of NI in crude extracts of the tissue. One solution is to rapidly remove AI from crude extracts by Concanavalin A chromatography [152]. Activity assays of NI in low AI cultivars show that the active enzyme is present in the grape pericarp (Table 2; [94]). In grape pericarp, at least five NI genes are transcribed. Transcript abundance is dependent on the gene in question and stage of development: transcripts of some genes are more abundant before ripening, whilst for other genes, the situation is reversed [79].

### 4.3. Acid Invertase

Transcripts arising from two grape VAI genes are present in the pericarp; however, those of *VvGIN1* are much more abundant. Large amounts of soluble AI activity are present throughout development (Table 2; [7,11,103,153]). In some plant tissues, the AI polypeptide is present as fragments (i.e., the invertase polypeptide is broken at one or more positions *in vivo*); however, the function is unknown [86]. The predicted molecular weight of the polypeptide produced by both of the VAI genes is 61 kDa [103]. The VAI polypeptide in pericarp extracts of some grape cultivars is largely intact, whilst in others, it is not [154,155,156,157]. In the flesh of other fruits, the bulk of the enzyme can either be present in the unfragmented form (cherry; [96]) or the fragmented form (tomato (*S. lycopersicon*]; [101]).

In grape pericarp extracts, a large proportion of soluble AI activity is due to VAI [153]. However, it is uncertain exactly what proportion is due to CWAI [11]. This is because it is possible that a proportion of the activity in the soluble fraction is due to CWAI that was solubilised, and the reverse can apply to the particulate fraction [97,105]. CWAI transcripts and protein are present in the flesh and skin of grape pericarp throughout development [75,77,158]. Significantly, Sarry et al. [64] found that a 31 kDa CWAI fragment was almost as abundant as VAI polypeptide in the ripe pericarp.

Immunohistochemistry showed that CWAI protein was present in diverse locations in grape pericarp [7]. However, these results have been considered artefactual by some workers; because the antibody used was considered to be non-specific [10]. This raises the question of whether these results are real or an artefact. Famiani et al. [7] used invertase antibodies that were a kind gift of Arnd Sturm (FMI, Basel, Switzerland). The CWAI antibody was raised to the deglycosylated protein from carrot [159], and the antibody to VAI was raised by Sturm’s group against the terminal 2/3 of carrot VAI isoform I that had been over-expressed in *E. coli*. The VAI antibody has a specific and very strong cross-reaction with grape berry VAI [7,137]. The CWAI antibody has been shown to have high specificity for CWAI from a range of species [160,161,162], and if any cross-reaction was found with the vacuolar form, it was weak [163]. Further, the observation that the antibodies raised against CWAI and VAI labelled different cell types in grape berry sections [7] is a strong indication that the antibodies recognised different proteins. One could question whether other aspects of the immunohistochemical methods used by Famiani et al. [7] gave rise to artefactual results. This is unlikely because when identical methods that employed the CWAI antibody were used in the same laboratory [22], the same locations in maize kernels were found to those established by others [161]. Thus, it appears unlikely that the localisation studies of CWAI and VAI in grape berries by Famiani et al. [7] were artefactual.

### 4.4. Sucrose Cycle

**Figure 2:**
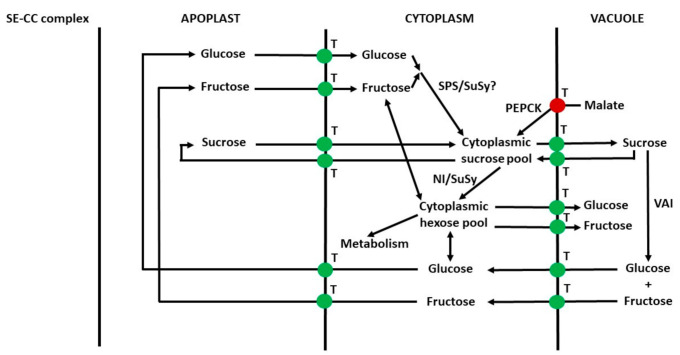
The sucrose cycle consists of the breakdown and resynthesis of sucrose from its breakdown products. Sucrose has a pivotal role in cell metabolism; it can be imported, exported, accumulated, broken down and resynthesised in different cell compartments. This allows a multifaced control of sugars concentrations and fluxes in the cell compartments, thereby modulating the various sugar functions in a complex metabolic network. Abbreviations: NI = neutral invertase; PEPCK = phosphoenolpyruvate carboxykinase; SE-CC = sieve element-companion cell; SuSy = sucrose synthase; SPS = sucrose phosphate synthase, T (green circle) = sugar transporter; T (red circle) = malate transporter; VAI = vacuolar acid invertase.

In sinks, a cycle of sucrose breakdown and resynthesis often occurs. This is termed the sucrose cycle (Figure 2; [108,164,165,166]).

The occurrence of the sucrose cycle in grape pericarp was established in the 1960s by ^14^C radiolabelling studies [167], in conjunction with some of the first studies of enzymes in grape berries [92]. Hawker [92] found that the activities of SuSy, SPS, sucrose phosphate phosphatase and AI were present in sufficient quantities to allow breakdown and resynthesis of all the sucrose as it was imported into the berry. A sucrose cycle was known to occur in the storage tissue of sugar cane, and it was thought (incorrectly; [168]) that sucrose (unlike glucose and fructose) was not transported across the plasma membrane of storage cells. Thus, sucrose was converted to glucose and fructose by CWAI to allow uptake [169]. It was unknown whether sucrose could be transported across the plasma membrane of grape pericarp cells, and the function of the sucrose cycle was uncertain [92]. Today, it is clear that in various plant tissues, the sucrose cycle plays a key role in coordinating sugar utilisation and accumulation and sugar concentrations in different subcellular compartments/apoplast [149,170,171,172]. The cycle involves sugar transport between subcellular compartments, and a number of enzymes and transporters are involved (Figure 2; [108,164,165,166]). In tomato pericarp, the sucrose cycle occurs both early in development (when sugars are transported from phloem to sink cells symplastically) and later in development (when transport is apoplastic) [63,149]. Similarly, there is evidence that the sucrose cycle occurs in grape pericarp both before and after veraison [92,167]. In grape pericarp parenchyma cells that accumulate sugars, it is possible that certain enzymes/transporters function largely in the sucrose cycle and not in the direct transfer of sucrose from the phloem to the storage cell vacuole.

### 4.5. Sucrose:Hexose Ratio in Fruits before Ripening

In the flesh of most fruits before ripening, the total content of sugars is low, as is the S:(G+F) ratio (Table 1). This low S:(G+F) ratio is associated with the presence of VAI and is a feature of many expanding cells. In these, it can contribute to increasing their turgor pressure [17,173,174,175]. In grape pericarp before ripening, organic acids, glucose/fructose and inorganic ions are quantitatively important osmotica [17,170,171,172]. In trichomes and stomata, all these compounds can contribute to determining turgor pressure changes, and their relative importance depends on various factors [176,177]. Turgor pressure can potentially affect both cell expansion and symplastic import of material, and both occur in the flesh of fruits before ripening [1,178]. In root tips, a turgor pressure gradient between the phloem and sink cells is an important regulator of symplastic bulk flow of liquid from the phloem [174]. In developing potato tubers, the S:(G+F) ratio, which could be altered by AI, might also play a role in the regulation of this flow [35]. Whether high turgor pressure assists cell expansion depends on the cell in question and its stage of development [174,175,178]. Cell wall properties can be the critical factor [174,179], and in ripening grape pericarp, cell expansion occurs at low turgor [37,180]. In many ripening fruits, this expansion at low turgor is made possible by changes in the structure of the cell wall during ripening [178].

### 4.6. Sucrose:Hexose Ratio in Fruits during Ripening

In many sink tissues, including grape pericarp, vacuolar S:(G+F) is inversely correlated with the abundance of VAI (Table 1 and Table 2; [43,86,94]. Indeed, reducing the abundance of VAI in tomatoes (*S. lycopersicon*) fruits by antisense technology greatly increases this ratio [181]. However, CWAI is also involved. There is evidence that the S:(G+F) ratio of the vacuole and apoplast are often correlated (See Section 2.5). Further, asymmetrically labelled sucrose studies show that apoplastic hexose is transferred directly to the vacuole in the tomato pericarp [41,62,63]. The hypothesis and evidence that invertase determined this ratio in fruits, including grapes, was presented more than 100 years ago [182].

In some fruits, SPS is also involved in determining S:(G+F). In melons, it appears that during ripening, sucrose synthesis from recently imported raffinose family oligosaccharides using SPS, combined with a decrease in VAI, are important in increasing S:(G+F) [183]. However, this correlation between SPS and S:(G+F) was not found in many other species [100], including grape [43,94]. Clearly, it is possible that in some fruit species, different mechanisms using SPS could potentially contribute to an increase in S:(G+F). For example, a decline in VAI during ripening, combined with a net efflux of vacuolar hexoses, their conversion to sucrose and its transfer to the vacuole.

In some wild fruits, the primary purpose of accumulating sugars is to impart an attractive taste so that animals disperse their seeds. The amount of sugar required is reduced by accumulating hexose; because the sweetness of one molecule of glucose plus one molecule of fructose is double that of one molecule of sucrose [150]. Thus, the construction cost of the fruit is decreased greatly by accumulating hexose, and in certain circumstances, this could be an evolutionary advantage. Potentially, this could be a primary reason why fruits such as grape accumulate hexoses.

## 5. Leaf Sugar Metabolism

Informative micrographs illustrating the structure of grape leaves are shown by Paczek et al. [184].

### 5.1. Photosynthesis

Young grape leaves are sinks, and the import of sucrose is likely symplastic [75]. When they approach 30–50% of their maximum surface area, they become a source [12,185,186]. Profound changes in transport processes, sugar contents, enzyme complement and polypeptide composition occur during this transition [54,75,132,187]. Grape leaves use the C3 photosynthetic pathway [188]. Although photosynthetic CO_2_ fixation can be about 20 µmol s^−1^ m^−2^, it is usually much lower and is dependent on factors such as light intensity, time of day, leaf age, sink demand and water deficit [14,32,189]. Dayer et al. [189] found a large decrease in the rate of CO_2_ fixation commonly occurred after around midday. Hunter et al. [32] reported values of about 2–6 µmol s^−1^ m^−2^, which were dependent on the time of day and date during the season. Measurements of photosynthesis in vines with differing ratios of leaf area to fruit level indicate that photosynthesis is often sink-limited [14,190].

### 5.2. Sugar Content

The bulk of the non-structural carbohydrate content of grape leaves consists of sucrose, glucose and fructose together with starch (Table 1; [55,189,191,192]). Sugars are a predominant osmoticum in grape leaves [193]. These sugars arise from triose phosphate produced by photosynthesis. Triose phosphate is either converted to starch in the chloroplast or exported to the cytosol and converted to sucrose. Sucrose is also synthesised from products of starch degradation (i.e., glucose and maltose) that are exported from the chloroplast. Sucrose synthesis in leaves utilises the SPS pathway [192,194,195,196]. Sucrose can be exported or transferred to the vacuole in which it can be hydrolysed to glucose and fructose by VAI [197].

Starch, sucrose, glucose and fructose contents are dependent on factors that can include: time during the diurnal cycle, leaf position, crop level/sink demand, time during the season and vine water status [55,189,191,192,198]. Some studies found little change in soluble sugar content during the diurnal cycle [191,192,199], whilst others found larger changes [32,199,200]. In one study, glucose and fructose both increased at least two-fold during the night [200]. Hunter et al. [32] found sucrose content increased 1.5–3-fold during the day. Two studies reported starch content g^−1^ FW decreased about 50% during the dark period [191,192]. Further, nighttime starch decrease depends on: time during the season, leaf:fruit ratio and water deficit [14,189]. Before veraison, a 1.5–2-fold increase in starch content occurred during the day, and after this, there was no increase [14]. Decreasing the number of leaves on the vine prevented this increase in the remaining leaves before veraison [14]. Contents of soluble sugars and starch are affected by leaf export capability, and both increased in leaves on girdled shoots [201]. Leaf soluble sugar:starch content varies, and ratios of 20 at veraison and 5–10 at harvest have been reported [189]. By contrast, much lower ratios have been reported [191,192,199]. For example, the ratio varied from about 0.4–2, which was dependent on time during the diurnal cycle and leaf nitrogen status [192]. Leaves of many plants accumulate starch and soluble sugars during the day, which are then exported at night [194,195,202,203]. A similar situation can occur in grapevine [199], and the data of Chaumont et al. [191] and Chen and Cheng [192] are in agreement with this.

### 5.3. Acid Invertase

Grape leaves contain large amounts of AI activity throughout development, and the amount g^−1^ FW can be comparable to the pericarp (Table 2: [54,103,204]). VAI and CWAI transcripts are present, and AI activity is present in the soluble and insoluble fractions of extracts [54,75,103]. Large amounts of AI activity are present in the leaves of most plant species, and despite around 100 years of study, its function remains largely a mystery [110,197,205,206,207].

Huber [207] found that in the mature leaves of some plant species (as in most fruits), the amount of AI activity present is inversely proportional to sucrose content. However, this is not true for many species [197]. Indeed, grape leaves contain high amounts of sucrose and AI activity (Table 1 and Table 2; [54,103,204]). In the leaves of many plants (unlike fruits), a large proportion of the sucrose content is not located in the vacuole or apoplast, where AI is located [1,24]. The over-expression of yeast AI throughout either the apoplast or vacuolar compartment of leaves perturbs their function [208,209]. This suggests that naturally occurring AI is localised in specific tissues, and/or its activity is subject to regulation. There is strong evidence that supports both suggestions [110,197]. Bonfig et al. [110] provided evidence that a proportion of AI activity might not be active in planta because it is bound to invertase inhibitor protein. Then upon extraction, or in response to pathogen attack/wounding, AI is activated by its release from the inhibitor. It was suggested that this explained the presence of AI in mature leaves (i.e., it is part of the plant defence system and often inactive) [110]. However, this is not consistent with the tissue localisation of AI [197] or with the observation that reducing either CWAI or VAI in carrot leaves by antisense technology produces phenotypes that are not related to an impaired ability to deal with pathogen attack or wounding [162].

AI is localised in certain tissues/cells in leaves, and its functions can only be understood if this is considered [197]. However, because the functioning/physiology/biochemistry of many of these tissues/cells is not well understood, the function of AI is usually not apparent. In the epidermal layer, VAI has been implicated in stomatal movement; and its role is likely in the alteration of vacuolar osmotic potential [210,211]. In photosynthetic cucumber cotyledons, CWAI is abundant in trichomes [197]. Trichomes are linked to other cells by plasmodesmata [212], and a possible function is in altering their turgor pressure, which could modulate exchanges of liquid. A second possibility is that, as in tomato trichomes [213], it is invertase modified to catalyse a different reaction. AI is abundant in the vasculature of many leaves, and in photosynthetic cucumber cotyledons, CWAI is abundant around the xylem vessels [197]. As in the xylem vessels of other grapevine organs, two potential and related functions are possible. First, there is evidence that increased xylem-vessel hexose content brought about by xylem-located CWAI might play a role in embolism management. The latter might play a key role in regulating hydraulics in response to drought/frost stress [214,215]. Second, CWAI could function in osmoregulation associated with regulating liquid flows associated with the vasculature. Water for phloem flow is provided by xylem flow [216]. Tomato leaves that contained decreased CWAI had an increased phloem export rate [217], which is not inconsistent with CWAI playing a role in regulating liquid flows.

In both grape leaves and berries, an increase in abundance of VAI and/or CWAI and various HTs is associated with drought and the abundance of abscisic acid (ABA; a hormone associated with drought stress) [156,218,219]. Embolisms occur in the xylem of grape leaves/petioles only after prolonged periods of water deficit [220,221], and it is possible that alterations in liquid flow contribute to water management that plays a role in embolism-avoidance.

**Figure 3:**
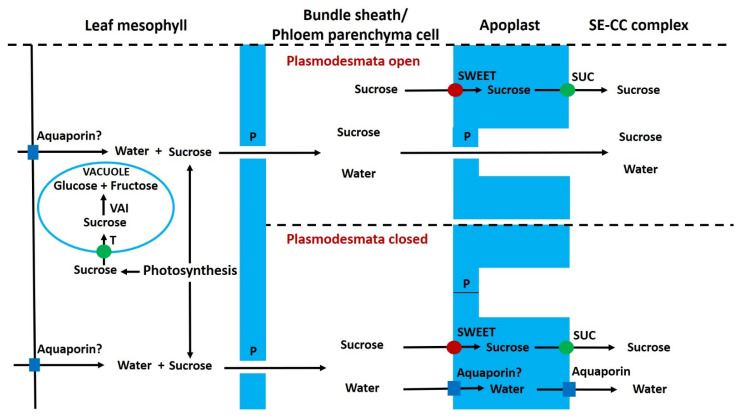
Hypothetical scheme depicting mixed loading of sucrose in grape source leaves. Briefly, sucrose is synthesised in the leaf mesophyll cells and then moves symplastically via plasmodesmata into the phloem parenchyma cells. Then, sucrose can enter the SE-CC complex either directly via plasmodesmata or can enter the apoplast via a SWEET and then enter the companion cells via a SUC. The latter is the only way if plasmodesmata are closed. The dashed line divides the situations of plasmodesmata open (upper part of the figure) or closed (lower part of the figure). Aquaporin water channels enable a rapid osmotically driven bulk flow of water. Abbreviations: P = plasmodesmata; SE-CC = sieve element-companion cell; SWEET = sucrose will eventually be exported transporter; SUC = sucrose transporter; T = sugar transporter; VAI = vacuolar acid invertase.

In mature leaves, AI is present in mesophyll cell vacuoles [152,197]. These contain the bulk of the leaf glucose and fructose content [18,24,30]. Tomato leaves lacking VAI contained almost no glucose/fructose, and photosynthesis was not reduced [222]. The data of Scholes et al. [222] show that in leaves lacking VAI, there was about a three-fold reduction in the amount of sucrose exported at night. In addition, the change in leaf soluble sugar contents is consistent with a decrease in both whole leaf and vacuolar sugar contents. Thus, the presence of VAI appears to increase vacuolar sugar content and alter the diurnal pattern of sucrose export. There is evidence of both osmotic and turgor pressure gradients directed towards minor veins and that VAI is at lower abundance in the bundle sheath compared to the mesophyll [31,223,224]. Potentially, this turgor gradient could bring about a bulk flow of liquid from the mesophyll to the bundle sheath/vascular parenchyma (Figure 3; [224]). In sink leaves, VAI could have the reverse effect. In sink leaves, the import of sucrose is symplastic [225], and as in some other sink tissues [35], VAI, could potentially increase sink cell turgor and decrease symplastic import.

## 6. Transport of Sugars within and between Organs

### 6.1. Pre-Phloem Transport and Phloem Loading of Sucrose in Leaves

Sucrose is synthesised in the cytosol of mesophyll cells and is thought to move towards the phloem in the symplast. Although what drives this movement is open to debate, it appears that bulk flow can be used [36,224]. Water required for phloem flow out of the leaf is provided by xylem flow [216]. The pathways of water flow in leaves are complex and subject to debate [226,227]. However, there is evidence that under most conditions, the apoplastic and gas-phase pathways are likely to account for the bulk of movement outside the bundle sheath [228]. The concentration of sucrose in the leaf bulk apoplast is low, and sucrose in the apoplast is carried by transpirational flow away from the phloem [34].

The entry of sucrose into phloem sieve elements/companion cells (SE/CC) is termed phloem loading. Depending on the species, phloem loading can be apoplastic (sugars enter the apoplast before loading), symplastic (sugars do not enter the apoplast before loading) or mixed (a combination of apoplastic and symplastic routes) [30,224,229]. Plant species that have a large number of plasmodesmatal connections between bundle-sheath/vascular parenchyma and the SE/CC can potentially use symplastic loading. Most woody plants such as grapevines have such plasmodesmatal continuity and can potentially use symplastic/mixed loading [30,75,230]. However, the relative contributions of apoplastic and symplastic loading in grape are unknown [130,176,231].

Apoplastic loading utilises SWEET facilitators that transport sucrose out of the symplast in the vicinity of the phloem, SUTs transport sucrose into the SE/CC and water moves by osmosis via aquaporins into the SE/CC (Figure 3; [26]. The polymer trap model has been used to account for the loading of oligosaccharides in symplastic loaders [229]. However, this model does not explain how symplastic loading can occur in species, such as grape, that do not transport oligosaccharides. A mixed loading model to account for symplastic loading of sucrose was provided by Voitsekhovskaja et al. [30] (Figure 3). Thus, a turgor pressure gradient drives bulk flow from the mesophyll to the bundle-sheath/vascular parenchyma. Apoplastic sucrose loading by SUTs increases the solute content of SE/CC. The hydrostatic pressure of the bundle sheath/vascular parenchyma can be similar or higher than that of the SE/CC (because the latter is not in the water-potential equilibrium), and this drives a bulk flow of liquid via plasmodesmata into the SE/CC [30]. Plasmodesmata are sensitive to differences in turgor pressure, and if this difference is too high, they close. An elegant mechanism that describes the mechanism by which plasmodesmata rapidly open or close has been described by Park et al. [232]. According to this model, the difference in pressure across the plasmodesmata causes the dumbbell-shaped ER-desmotubule complex to be displaced from its equilibrium position resulting in plasmodesmatal closure. In conclusion, the relative contributions of the symplastic and apoplastic loading routes could change with leaf water status, various environmental factors and stages of development [30].

Whatever the loading mechanism used, sucrose moves to sink tissues by bulk flow through the phloem sieve elements, and this is driven by a turgor pressure gradient between source and sink. This gradient is largely produced by the influx of water into the SE/CC in the source and its efflux in the sink. A reduction in efflux in the sink increases the turgor pressure of SE/CC at sites of unloading. This pressure change can be transmitted within minutes to the SE/CC in the leaf, which can contribute to the inhibition of both phloem loading and photosynthesis [190,233].

### 6.2. Phloem and Xylem Sucrose Contents

The sugar content of xylem-liquid from grapevine stems appears to be <0.5 mM [234,235]. The phloem-sucrose concentration is uncertain; because it is difficult to obtain pure phloem liquid from grapevines [236]. Pedicel exudates have been assumed to be pure phloem liquid, and their sucrose concentration was <50 mM [158,236]. This is around an order of magnitude lower than phloem-liquid soluble carbohydrate contents from a range of other plants, determined using the accurate aphid stylet technique [24,237,238]. Zhang and Keller [236] pointed out that a concentration of <50 mM is very low for phloem functioning. Phloem sugar concentration can also be calculated by the subtractive method. This entails preventing phloem flow into the berry by girdling: phloem flow is the difference in flow before and after girdling. A comparison of phloem flow and dry matter accumulation enables phloem liquid dry matter content to be calculated [239,240]. Thus, it can be calculated, from the rates of phloem flow and sugar accumulation/utilisation [10,134,241], that the sucrose concentration in the phloem liquid entering the berry can be about 200–600 mM. Nevertheless, it is possible that disruption of the phloem alters xylem flow: by preventing xylem backflows from the berry. Consequently, phloem-sucrose concentration could be low because excess phloem water could potentially be exported to the plant in the xylem [236,242].

The sugar concentration in exudates collected from peach was also found to be <50 mM [243], which is an order of magnitude lower than determined by the subtractive method [239]. However, in peaches, the aphid stylet technique has been used to obtain phloem liquid from different parts of the plant, and sugar concentration was 700–800 mM [24,244]. Early studies of cucurbits that measured the composition of sap exuding from cut stems led to the idea that their phloem sugar concentration was low. However, it is now clear this was incorrect because the liquid obtained by the exudation technique was not pure phloem liquid. The actual concentration of sugars in their phloem liquid is around 1M [245,246,247]. Thus, it is possible (or very likely) that the exudates obtained from the grape are not pure phloem liquid, and for this reason, their sucrose concentration is low.

### 6.3. Phloem and Xylem Flows into the Fruit

Although some sugars are produced by pericarp photosynthesis, almost all the water and solutes required by the berry are imported into the vasculature; and the bulk of sugars are imported as sucrose in the phloem [10,11]. The vasculature enters the berry at its base, and a proportion forms a central stand that runs from the base to the top of the berry, whilst the remainder gives rise to the peripheral network, which is located in the outer mesocarp. The peripheral network has branches that ramify into the pericarp in an inward direction [10,158,248].

Before the start of ripening, xylem-liquid input is much greater than phloem-liquid input; however, during ripening, the situation is reversed [10,236,241,249,250,251]. Similar observations have been made concerning liquid inputs into the fruits of sweet cherry [240]. By contrast, in peach fruits, xylem-liquid input is higher throughout development [239]. A widespread view (although not universally accepted for all cultivars; see [252] is that the xylem supply to grape berries remains conductive throughout development [198,236,253]. It has been suggested that an increase in apoplastic solutes in grape pericarp results in the hydrostatic pressure of the apoplast and xylem being similar, which could prevent, or reverse, xylem flow [242,248]. In grape berries, CWAI is present in xylem-vessel walls in both the pericarp and developing seed [7]. CWAI is also present in the xylem in other plant tissues [197,254,255]. The presence of CWAI in the vicinity of xylem vessels could increase apoplastic solute content by hydrolysing sucrose and thus contribute to an increase in hydrostatic pressure because water would be drawn into the apoplast by osmosis. As suggested by Keller et al. [242], water and sucrose could arise from vascular parenchyma cells and the phloem. Clearly, mineral ions could also be involved. The involvement of solutes in regulating xylem flows in plants is well established [256,257], as are transfers of water and solutes between xylem and phloem [258,259]. VI in both xylem and phloem vascular parenchyma cells could potentially play a role in creating turgor pressure gradients that drive symplastic movement of solutes [260]. This could be important under conditions that alter transpiration or water inputs into the berry, such as drought and rainy weather. During the latter, it is possible that liquid exchange between xylem-phloem creates liquid flows that distribute imported solutes from the phloem to mesocarp parenchyma cells. Sugar metabolism in the vascular parenchyma cells could be somewhat similar to that in the storage parenchyma cells and involve the sucrose cycle (Figure 2). Starch acts as a store of carbon in the vascular parenchyma cells of grapevines [261]. Such starch would interface with the sucrose cycle through glucose.

### 6.4. Post-Phloem Transport in the Seed

In grape seed coats, the palisade layer functions to distribute phloem-unloaded assimilates to the enclosed developing storage tissues [5]. Numerous plasmodesmata connect the cells of the palisade layer and are thought to facilitate symplastic movement. CWAI is particularly abundant in the palisade layer [5,7]. It is necessary to maintain a suitable turgor pressure gradient between the phloem and the cells through which post-phloem symplastic flow occurs, and thus, CWAI could function in adjusting the turgor of the palisade cells (by increasing the apoplastic concentration of solutes and hence drawing water out of the cell by osmosis) [1,262]. The involvement of CWAI in such a process is not without precedent [35,260,263]. Underlying the palisade layer are transfer cells, and their large cell wall area must assist in the transport of sucrose/hexose to the enclosed storage tissues [5]. Presumably, as in Arabidopsis [264], SWEET facilitators are located in the plasma membranes of these cells and are involved in the release of sucrose into the apoplast that bathes the developing storage tissues.

### 6.5. Phloem Unloading in the Pericarp

Phloem unloading is the movement of materials out of the SE/CC. This movement can be through plasmodesmata (symplastic unloading) or across the plasma membrane into the apoplast (apoplastic unloading). In grape pericarp, symplastic tracers passed from the phloem into the symplast of the flesh before ripening but not after. This suggests symplastic unloading is predominant before ripening and apoplastic unloading afterwards [158]. Nevertheless, the situation might not be so straightforward; because more than one switch between apoplastic/symplastic unloading can occur in tomatoes, and in plums, environmental conditions can also bring about switches [123,265,266].

In grape pericarp, a number of changes might be involved in bringing about this switch in the unloading mechanism. One is the closure of plasmodesmata [158]. As in some other tissues [30], it is possible that changes in turgor pressure between the SE/CC and adjoining cells bring about closure. Indeed, in grape pericarp, there is a decrease in turgor pressure of the parenchyma cells in the flesh at the onset of ripening [267], and this decrease (about 0.2 MPa) is of the magnitude known to rapidly bring about plasmodesmatal closure [30,232]. However, it is unknown whether the decrease in turgor pressure occurs before the switch to apoplastic loading [267]. A second factor is increases in abundance/flux through phloem-located SWEETs and aquaporins. Osmoregulation could be a reason why this switch from symplastic to apoplastic unloading is necessary. Thus, the accumulation of large amounts of sugars in the parenchyma cells during ripening requires a large increase in their apoplastic concentration; in order for the fruit to soften [37].

**Figure 4:**
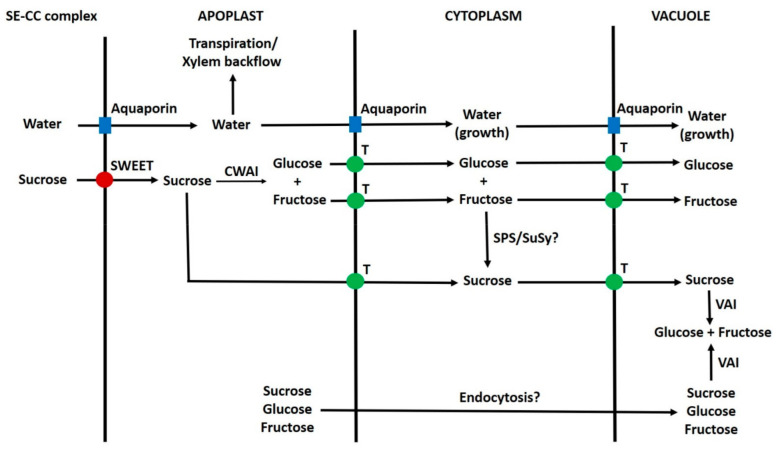
Sucrose transport and associated metabolism in grape pericarp during ripening. Sucrose exits the SE-CC complex via a SWEET, then a proportion of sucrose is hydrolysed by CWAI. Sugars then move through the apoplast, and this movement can be brought about largely by transpiration. Sugars can potentially enter parenchyma cells and move to the vacuole by several pathways. The contribution of each pathway is unknown. Aquaporin water channels enable a rapid osmotically driven bulk flow of water. Abbreviations: CWAI = cell wall acid invertase; SE-CC = sieve element-companion cell; SuSy = sucrose synthase; SPS = sucrose phosphate synthase; SWEET = sucrose will eventually be exported transporter; T = sugar transporter; VAI = vacuolar acid invertase.

Photosynthesis in grapevines is often sink-limited [14,190], and this implies phloem unloading is not constant. Indeed, large changes in berry phloem-input occur during the diurnal cycle, and during ripening, the input can be much higher during the day [241,251]. This suggests that unloading is regulated, and it is likely that changes in flux through SWEETs and aquaporins play a role (Figure 4). In both potato tubers and peach flesh, SUTs are localised in the phloem and could contribute to regulating the turgor pressure of SE/CC by transferring apoplastic sucrose into them [268,269]. A grapevine SE/CC-localised HT with high specificity for glucose [121] could function similarly, and as in other plants [145], glucose could be converted to sucrose within the companion cell. Turgor pressure changes in sink phloem are thought to be involved in coordinating phloem loading and unloading [190,233].

### 6.6. Symplastic Post-Phloem Transport in the Pericarp Before Ripening

The movement of material after leaving the phloem is termed post-phloem transport. Both bulk flow and diffusion can potentially contribute to symplastic post-phloem transport [270]. In roots, at least, it appears that diffusion is too slow to account for the rate of sugar import, and symplastic bulk flow, driven largely by the higher hydrostatic pressure of the phloem, is used [271]. Grape pericarp parenchyma cells show a mosaic of development. Early in development, clusters of cells contain crystalloid inclusions, whilst other clusters do not. It appears that these crystals usually disappear as the cell develops, and CWAI is localised in cells possessing crystals and VAI in those without [7]. In developing potato tubers, it is hypothesised that CWAI might lower the turgor pressure of storage cells, which would favour symplastic flow into them, whereas VAI would do the reverse [35]. In grape pericarp, if a proportion of post-phloem symplastic transport is by bulk flow, it is possible that the AIs function as proposed for potato tubers. The contents of sugars in the bulk symplast and apoplast of grape pericarp before ripening [37] are not inconsistent with this possibility. However, information regarding apoplastic/symplastic sugar concentrations in localised regions of the pericarp is required (e.g., in clusters of crystal-containing cells). Further, these concentrations could change during the diurnal cycle, as do xylem and phloem-flows [241,251].

### 6.7. Apoplastic Post-Phloem Transport in the Pericarp during Ripening

In peach flesh throughout its development, apoplastic bulk flow of liquid is driven by transpiration [239,272]. In grape pericarp, transpiration occurs throughout development [236,273,274], and it is likely that associated liquid flows make a large contribution to the post-phloem movement of sugars through the apoplast to the parenchyma cells. Indeed, in ripening berries under some environmental conditions, phloem-input and transpiration are quantitatively similar, as are the patterns of change during the diurnal cycle [241,251]. Using values of transpiration from the literature [10,241,251], and assuming the liquid-filled apoplast is around 5–10% of tissue volume, then during ripening, all the apoplastic liquid could be displaced by transpiration-driven flow in a day under many environmental conditions.

### 6.8. Phloem Unloading, Apoplastic Sugars and Invertase

In the flesh of commercially grown hexose-accumulating tomatoes (*S. lycopersicon*), asymmetrically labelled sucrose studies showed that a large proportion of sucrose unloaded from the phloem is hydrolysed by CWAI, and then both hexose and sucrose enter sink cells [41,62,63]. The relative contributions of cell wall-located AI in the SE/CC and in parenchyma cells to this hydrolysis are unknown [63]. In ripening grape pericarp, CWAI is present in the SE/CC and its abundance increases at the onset of ripening [158]; however, localisation data regarding its presence in parenchyma cells during ripening are inconclusive [7]. It is generally assumed that SE/CC-located CWAI plays an important role in phloem unloading. However, this potential role is open to debate [224], and some possible roles include: increasing phloem sucrose efflux by enhancing sucrose transporter efficiency, drawing water out of the phloem by an osmotic effect and thus increasing phloem flow from the source, signalling and increasing the rate of sucrose diffusion between the phloem and sink cells (e.g., [86,111,275]. At least in some sucrose-accumulating tomato species (*S. chmielewskii* and *S. habrochaites*), the bulk of the sucrose that is transported from the phloem to the parenchyma cell vacuoles is not hydrolysed during unloading/transit [62,63]. In these fruits, AI abundance is low, and transcripts for LIN5 are at low abundance or not present [62,63,276]. LIN5 is the CWAI present in phloem sieve elements of the fruits of *S. lycopersicon* [265]. This raises the question of the importance of CWAI in phloem unloading. It is questionable whether phloem unloading is made more efficient by the participation of CWAI because the ripe fruits of *S. chmielewskii* and *S. habrochaites* contain around double the mass of sugar g^−1^ FW compared to the fruits of many *S. lycopersicon* cultivars [51,62,63,73,276]. An alternative function for phloem-located CWAI is that it contributes to the hydrolysis of unloaded sucrose, which plays a role in the osmoregulation of the storage cells. Indeed, CWAI has been implicated in this in grape pericarp [38,267]. Such a role for CWAI need not be restricted to apoplastic unloading from the phloem, and it is possible that such a role is widespread in sinks (e.g., developing seeds) in which sucrose is released into the apoplast.

### 6.9. Regulation of Apoplastic Sugar Concentration

In the pericarp, both before and during ripening, large inputs of xylem-fluid can occur (often at times when phloem-input is low), and these xylem inputs depend on environmental conditions and time during the diurnal cycle [241,251]. Similarly, in peaches, large xylem inputs occur throughout ripening [239]. Thus, a mechanism is required to avoid dilution of apoplastic solute concentration, and this likely involves the transport of sugars out of the cell in conjunction with the sucrose cycle (Figure 2; See Section 4.4). Indeed, asymmetrically labelled sucrose studies in tomatoes show that hexose absorbed by the cell can be converted to sucrose, which is then broken down, and hexoses can then re-enter the apoplast [63]. The cytoplasm occupies around 1% of cell volume in ripe berries [29], and the liquid-filled apoplast likely occupies around 5–10% of tissue volume. Apoplastic sugar concentration in ripening pericarp is around 800 mM hexose [37]. Thus, if all this hexose was displaced, this would correspond to around 7–15 mg hexose g^−1^ FW pericarp. If sugar content in the cytoplasm is similar to that in the apoplast and vacuole (See section ‘Compartmentation of Sugars in Pericarp Parenchyma Cells’) then the cytoplasm sugar content would be around 1.5–3 mg g^−1^ FW pericarp (depending on the ratio sucrose:hexose). Therefore, a considerable flux of sugars from the vacuole to the apoplast could be required, which would have a major effect on cytoplasmic sucrose metabolism (Figure 2). Clearly, parenchyma cells near the xylem input would need to release sugars, and those nearer to where the water was lost from the berry would need to absorb the sugars. Thus, regulating the apoplastic sugar concentration could have a major impact on fruit sugar metabolism.

### 6.10. Apoplast to Vacuole Transport of Sugars in Parenchyma Cells

A simplified scheme depicting the movement of sugars from the plasma membrane to vacuoles of grape pericarp parenchyma cells is shown in Figure 4. The relative contributions of each of the potential routes are unknown in grape and other fruits. As in tomatoes, the contributions of different transporters to the passage of sugars across the plasma membrane and tonoplast are unknown. However, in both grape and tomato SWEETs, SUTs and HTs might be involved [11,41,76,124,277,278]. The situation is complicated by the occurrence of the sucrose cycle (Figure 2), and some transporters could function primarily in this. Further, some transporters could be localised in the vasculature [121]. There is evidence from asymmetrically labelled sucrose studies in tomatoes that a small proportion of hexose is converted to sucrose during transport across the cytoplasm [63]. Perhaps an understated complication is the presence of large amounts of glucose and fructose in the cytosol, which is associated with their transport from the apoplast to the vacuole. These sugars could potentially be phosphorylated by hexokinase and fructokinase, respectively [279], and the question arises as to why they are not. In certain sink tissues, including the flesh of citrus fruits, there is evidence that sugars can be transported from apoplast to vacuole in vesicles by endocytosis/pinocytosis or by a close association of the plasma membrane and tonoplast [114,115,280]. Whether endocytic transport of sugars occurs in grape pericarp is unknown [11]. However, the occurrence of endocytosis would perhaps not be surprising; because vesicular transport is involved in the transport/metabolism of carbohydrates such as cell wall constituents and fructans [281,282,283]. Endocytosis could be one explanation as to how the presence of large amounts of hexose in the cytoplasm of fruit parenchyma cells does not result in the over-production of hexose-phosphates (because glucose/fructose and hexokinase/fructokinase would be in a different compartment). Indeed, evidence consistent with this was obtained in developing potato tubers [131]. A second possibility is that regulation of hexokinase/fructokinase activity prevents this phosphorylation.

### 6.11. Summary of Transport of Sugars from the Phloem to Vacuoles of Sink Cells

Based on the considerations presented in this review, a simplified scheme depicting sucrose transport from the phloem to vacuoles of grape pericarp parenchyma cells during ripening is shown in Figure 4.

**Figure 5:**
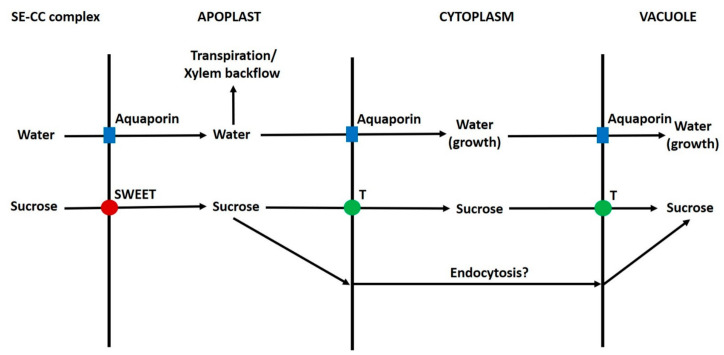
Sucrose transport and associated metabolism in sucrose-accumulating tomatoes. Sucrose exits the SE-CC complex via a SWEET, and then it moves through the apoplast largely by bulk flow, which can be largely generated by transpiration or, potentially, by xylem backflow. Sucrose can enter the parenchyma cells of the flesh and into the vacuole via transporters or, possibly, by endocytosis. Uncertainty about the occurrence of endocytosis is indicated in the figure by the question mark. Aquaporin water channels enable a rapid osmotically driven bulk flow of water. Abbreviations: SE-CC = sieve element-companion cell; SWEET = sucrose will eventually be exported transporter; T = sugar transporter.

In fruits of the tomato *S. lycopersicon* [41,63], it is likely that a large proportion of the sucrose unloaded from the phloem is hydrolysed in the apoplast by CWAI, and in grape pericarp, the situation could be similar. Sugars move through the apoplast to parenchyma cells by a transpiration-driven bulk flow of liquid. Hexose and sucrose enter parenchyma cells and are transferred to the vacuole, in which sucrose is hydrolysed by VAI. For comparison, a scheme depicting the situation in sucrose-accumulating tomato species *S. chmielewskii* and *S. habrochaites* is shown in Figure 5 (this is based on the considerations presented by: [41,62,63].

## 7. Acid Invertase Osmoregulatory System

The information given in this review suggests that AI has a diverse and widespread role in osmoregulation and associated transport processes in plants. Further, although many plants transport sugar alcohols or raffinose family oligosaccharides in the phloem, sucrose is co-transported [24,30]. It is plausible that a primary factor responsible for the use of sucrose as a transport sugar is: osmotic effects arising from its hydrolysis by AI are an integral part of many transport and osmoregulatory processes. AI plays a role in diverse processes [284]; however, an underlying role in many of these could be osmoregulation. The hydrolysis of sucrose increases solute concentration, and the reverse is true if it is converted to starch or fructan. Fructans are soluble fructose oligomers/polymers that are usually located in the vacuole, and in some plants, are used as an alternative to starch storage [285]. Clearly, in any situation, the storage of starch/fructan, as opposed to sucrose, will have osmoregulatory consequences. The fructan-metabolising enzymes are modified AIs that have evolved independently on a number of occasions from AIs (very small sequence changes are required) [285]. Thus, in one sense, the fructan-metabolising enzymes can be viewed as modified AIs that function to decreases solute content rather than increase it.

## 8. Conclusion and Future Perspectives

An impressive understanding of sugar metabolism in grapevines has been achieved by the dedicated work of a large number of researchers over a period spanning three centuries. However, the subject is extremely complex, and many questions remain unanswered. To answer these questions over the coming decades will doubtless continue to require a multi-disciplinary approach. To understand many aspects of sugar metabolism in grapevines, its compartmentation between/within organs/tissues/cells must be better understood. Further, temporal changes in metabolism, such as those associated with alterations in liquid inputs into the berry [241,251], and the release of solutes from the vacuole [21,22], need to be taken into account.

In this context, the use of genome-editing techniques based on the introduction of a double-strand DNA (dsDNA) break in a target genome site can help in the understanding of sugar metabolism and signalling both by inducing gene knockout or making possible a gene replacement. The more versatile genome-editing tool is the clustered regulatory interspaced short palindromic repeats (CRISPR)-Cas systems [286]. Identified as conserved mechanisms against viral invasions in bacteria, CRISPR-Cas systems have three components: a protein with nuclease activity (e.g., Cas9), a single guide RNA (sgRNA) needed to guide the Cas protein to the target sites, and a protospacer adjacent motif (PAM), a target site tag that is a short sequence upstream the complementary DNA strand. The sgRNA-Cas complex scans the genomic DNA identified the complementary DNA sequence, and the Cas protein induces a dsDNA cleavage at a specific position, thus activating the cell DNA repair pathways. In plants, the first reported genome-editing application using CRISPR/Cas systems dated back to 2013 on two model plants: *Arabidopsis thaliana* and *Nicotiana benthamiana* [287,288]. However, more recently, applications of genome editing in woody species have been reported [289]. In plants, the DNA sequence encoding for Cas and sgRNA(s) can be delivered into the host genome by two different methods: Agrobacterium-mediated transformation or biolistic transformation, which requires the integration of T-DNA into the host genome [290]. Alternatively, protoplasts transient transformation and regeneration allow the direct delivery of ribonucleoproteins (RNPs) in plant tissues without genome-stable transformation, the development of methods that avoid the incorporation of transgenic DNA in the host plant genome allows the production of transgene-free genome-edited plants, technological improvements are necessary to overcome the important limitations existing in woody plant applications [291,292,293,294] due to explant types, age and physiological state of the tissues.

In grapevines, the application of the new genome-editing technologies can make important contributions to breeding new, improved varieties. In fact, these technologies may produce minimal and precise modifications in elite genotypes, preserving their whole genome without altering their genetic background, as occurs in traditional cross-breeding. In addition, the preservation of the wine typicity will be central to the acceptance of new varieties. Grapevine breeding is hampered by some intrinsic features of *Vitis vinifera*, such as being a perennial crop and the limitation that for evaluating important characteristics, there is the need for the vine to reach maturity and produce berries. These are also the reasons why the time for releasing a new variety to the market is longer compared to other important agricultural crops. Therefore, the grape industry is increasingly looking toward genome editing as a new opportunity to improve breeding for more sustainable viticulture as well as for producing healthier and more flavorful products. An important grape berry quality trait is the sugar content and composition. Classical breeding has already created varieties with low sugar content, producing wines with no more than 10–11% alcohol [295]. A reduction in wine alcohol content is indeed a further objective in grape improvement. The targeting of genes involved in sugar metabolism could open new ways for modifying the content of sucrose and alcohol in grapes, giving vine breeders the potential to control the levels of these important wine quality characteristics.

## Figures and Tables

**Table 1:** Typical contents of soluble sugars (mg g^−1^ FW) in the pericarp and leaves of grape and the flesh of some other fruits. Both Muscat Bailey A and Steuben are interspecific hybrids. Note apricot, cherry, peach and plum also accumulate sorbitol, and the contents of this compound for the cultivars given in this table are given in Walker et al. [1]. For grape berries, three stages of development termed I, II and III, can be distinguished. During stage I, the berry increases in size. During stage II, veraison occurs, and there is a slowing down of the growth rate. During stage III, ripening occurs and the berry increase in size. Ripening is accompanied by softening and compositional changes, such as a large decrease in organic acid and a substantial increase in the content of soluble sugars [9]. In this table, stage I–II correspond to the period up to veraison and stage III to the last part of the ripening phase.

Stage I–II				
	Sucrose	Fructose	Glucose	References
**Grape—Pinot Noir**				
Skin	<1	2	3	[21]
Flesh	<1	1	4	[21]
**Grape—Muscat Bailey A**				
Skin	<1	<1	3	[43]
Flesh	<1	<1	3	[43]
**Grape—Steuben**				
Skin	<1	<1	3	[43]
Flesh	<1	<1	3	[43]
**Apricot—common**	2	1	3	[44]
**Apricot—Japanese**	1.3	1.1	2.0	[45]
**Cherry—sweet**	˂1	9	23	[46]
**Peach**	3	13	14	[47,48]
**Plum—Japanese**	7	11	14	[49,50]
**Tomato**				
Hexose accumulator (*Solanum lycopersicon*)	0	13	12	[51]
Sucrose accumulator (*Solanum peruvianum*)	2	15	14	[51]
**Stage III-Ripe**				
**Grape (Pinot Noir)**				
Skin	8	66	72	[21]
Flesh	9	81	84	[21]
**Grape (Muscat Bailey A)**				
Skin	17	47	43	[43]
Flesh	2	50	54	[43]
**Grape (Steuben)**				
Skin	41	47	43	[43]
Flesh	29	63	65	[43]
**Apricot (common)**	65	6	18	[44]
**Apricot (Japanese)**	9.0	0.9	0.5	[45]
**Cherry (sweet)**	˂1	65	75	[46,52]
**Peach**	48	9	7	[47,53]
**Plum (Japanese)**	92	21	27	[49,50]
**Tomato**				
Hexose accumulator (*Solanum lycopersicon*)	<2	32	26	[51]
Sucrose accumulator (*Solanum peruvianum*)	73	16	8	[51]
**Grape—mature leaves**				
Riesling × Silvaner			7	[54]
Thompson seedless	7–10	4.5–7	5–8	[55]

**Table 2:** Typical approximate activities of sucrose synthase and the invertases (µmol g^−1^ FW h^−1^) in the pericarp, and in some cases skin and flesh, and leaves of grape and the flesh of some other fruits. Both Muscat Bailey A and Steuben are interspecific hybrids.

Stage I–II				
	Sucrose Synthase (Cleavage)	Neutral Invertase	Total Acid Invertase	References
**Grape**				
Muscat Bailey A	<0.25		180–370	[43]
Skin	0.2		70–160	[43]
Flesh	0.05–2		190–230	[43]
Steuben	<0.2		<4	[43]
Skin	0.2–0.5		2.5–5.0	[43]
Flesh	0.15–0.2		2.3–4.2	[43]
High sucrose cultivars	2.5	2	<10	[94]
Thompson seedless	1–3		110–350	[92]
**Asian pear**	2–10		8–33	[95]
**Cherry (sweet)**			22	[96]
**Grapefruit**				
Juice sacs	15	3.9	25	[97]
Major vascular bundles	12	2.0	49	[97]
Albedo of peel	4	1.6	91	[97]
**Kiwifruit**	40	3	8	[98]
**Peach**	10–20	2–12	6–30	[47,99]
**Strawberry**	6	2.5	25	[100]
**Tomato**				
Hexose accumulator (*Solanum lycopersicon*)	30		240	[101]
Sucrose accumulator (*Solanum chmielewskii*)	18		4	[101]
**Stage III—Ripe**				
**Grape**				
Muscat Bailey A	<0.4		310–360	[43]
Skin				[43]
Flesh				[43]
Steuben	<0.5		<2	[43]
Skin				[43]
Flesh				[43]
High sucrose cultivars	4	10	15	[94]
Thompson seedless	1.3		110–350	[92]
**Asian pear**	1–6		1–7	[95]
**Cherry (sweet)**			180	[96]
**Grapefruit**				
Juice sacs	0.1	0.6	0.4	[97]
Major vascular bundles	1.0	0.3	1.3	[97]
Albedo of peel	0.1	0.5	1.9	[97]
**Kiwifruit**	4	1	4	[98]
**Peach**	1–14	0–1.5	2–6	[47,99]
Increased from	5–25	<3	0	[100]
Large number of genotypes		average		
	3.6	1.8	1.5	[102]
		range		
	0–13	0–11	0–6	[102]
**Strawberry**	6	10	3	[100]
**Tomato**				
Hexose accumulator (*Solanum lycopersicon*)	1–3		1200	[101]
Sucrose accumulator (*Solanum chmielewskii)*	0.06–2.5		<0.3	[101]
**Grape young leaves**				
Riesling × Silvaner			90	[54]
Shiraz			322	[103]
**Grape mature leaves**				
Riesling × Silvaner			10	[54]
Shiraz			78	[103]
Several cultivars	1–5	2–6	5–35	[94]

## Data Availability

Not applicable.

## Supplementary Materials

The following is available online at https://www.mdpi.com/article/10.3390/ijms22157794/s1, Table S1: Genes encoding sugar transporters and those involved in sugar metabolism identified in the grape genome. In the table, the ID associated with the transcript name for the version 12X (at EMBL FN597015-FN597047, release 102) and version 12XC3 [296] are reported: to make easier the identification of genes mentioned in Figure 1 (see references in the last column). For each gene, the Vitis annotation (12X version) and best hits against the Arabidopsis genome sequences (TAIR, https://www.arabidopsis.org/Blast/, accessed on 10 May 2021) are also reported.
