# Supplementary material for: Sucrose Metabolism and Transport in Grapevines, with Emphasis on Berries and Leaves, and Insights Gained from a Cross-Species Comparison
Source: Int J Mol Sci. 2021 Jul 21;22(15):7794. doi: 10.3390/ijms22157794 (PMC8345980; doi:10.3390/ijms22157794)
Supplement: Supplementary file 1 [file ijms-22-07794-s001.zip › ijms-1240127-supplementary.pdf]

International Journal of
Molecular Sciences

MDPI

Supplementary Table S1. Genes encoding sugar transporters and those involved in sugar metabolism identified in the grape genome. In the table are reported the ID associated with the transcript name for the version 12X (at EMBL FN597015-FN597047, release 102), and version 12XC3 ([296]: to make easier the identification of genes mentioned in Figure 1 (see references in the last column). For each gene the Vitis annotation (12X version), and best hits against the Arabidopsis genome sequences (TAIR, https://www.arabidopsis.org/Blast/) are also reported.

Gene ID	transcriptName 12X	Transcript Name 12xC3	Vitis annotation	Best-hit-arabi-name	arabi-symbol	arabi-defline\	Reference
VvHT1	GSVIVT01003181001	Vitvi10g00358		AT1G11260.1	ATSTP1,STP1	sugar transporter 1\	[74,75]
VvHT2	GSVIVT01009024001	Vitvi18g00397	SUGAR TRANSPORT PROTEIN 5	AT1G34580.1		Major facilitator superfamily protein\	[74]
VVHT3	GSVIVT01001036001	Vitvi11g00611	SUGAR TRANSPORT PROTEIN 7	AT4G02050.1	STP7	sugar transporter protein 7\	[74]
VvSUC11	GSVIVT01009254001	Vitvi18g00584	solute carrier family 45, member 1/2/4 (SLC45A1_2_4)	AT1G09960.1	ATSUC4,ATSUT4,SUC4,SUT4	sucrose transporter 4\	[74,120]
VvSUC12	GSVIVT01020031001	Vitvi01g00959	SUCROSE TRANSPORT PROTEIN SUC3	AT2G02860.1	ATSUC3,ATSUT2,SUC3,SUT2	sucrose transporter 2\	[74,120]
VvSUC27	GSVIVT01034886001	Vitvi18g01315	SUCROSE TRANSPORT PROTEIN SUC1-RELATED	AT1G71890.1	ATSUC5,SUC5	Major facilitator superfamily protein\	[74,120]
VvTM1/VvHT6	GSVIVT01013414001	Vitvi18g00056	Sugar (and other) transporter (Sugar_tr) // Major Facilitator Superfamily (MFS_1)	AT4G35300.4	TMT2	tonoplast monosaccharide transporter2\	[74]

VvMT2	GSVIVT01023868001	Vitvi03g00247	MONOSACCHARIDE-SENSING PROTEIN	AT4G35300.1	TMT2	tonoplast monosaccharide transporter2\	[2,74]
VvSWEET10	GSVIVT01008595001	Vitvi17g00070	BIDIRECTIONAL SUGAR TRANSPORTER SWEET10	AT5G50790.1		Nodulin MtN3 family protein\	[76,78]
VsSWEET15	GSVIVT01000938001	Vitvi01g01719	BIDIRECTIONAL SUGAR TRANSPORTER SWEET10	AT5G13170.1	SAG29	senescence-associated gene 29	[76,78]
VvSuSy3	GSVIVT01028043001	Vitvi07g00353	SUCROSE SYNTHASE 2	AT4G02280.1	ATSUS3,SUS3	sucrose synthase 3\	[77]
VvSuSy4	GSVIVT01015018001	Vitvi11g00030	SUCROSE SYNTHASE 1-RELATED	AT3G43190.1	ATSUS4,SUS4	sucrose synthase 4\	[77]
SPS	GSVIVT01035882001	Vitvi04g00508	SF404 - GLYCOSYLTRANSFERASE // SUBFAMILY NOT NAMED	AT5G11110.1	ATSPS2F,KNS2,SPS1,SPS2F	sucrose phosphate synthase 2F\	[77]
VvNI1	GSVIVT01024105001	Vitvi03g00088	Beta-fructofuranosidase / Saccharase	AT4G34860.2		Plant neutral invertase family protein\	[77,79]
VvNI2	GSVIVT01034944001	Vitvi05g00164	Alkaline and neutral invertase (Glyco_hydro_100)	AT3G06500.1		Plant neutral invertase family protein\	[79]
VvNI3	GSVIVT01031267001	Vitvi14g00070	Alkaline and neutral invertase (Glyco_hydro_100	AT1G56560.1		Plant neutral invertase family protein\	[79]

VvNI4	GSVIVT01031374001	Vitvi06g01427	Alkaline and neutral invertase (Glyco_hydro_100)	AT5G22510.1	At-A/N-InvE,INV-E	alkaline/neutral invertase\	[79]
VvCWAI	GSVIVT01016869001	Vitvi09g00193		AT3G13790.2	ATBFRUCT1,ATCWINV1	Glycosyl hydrolases family 32 protein\	[75,77]
VvAI1	GSVIVT01018625001	Vitvi06g01427	beta-fructofuranosidase (E3.2.1.26, sacA)	AT1G62660.1		Glycosyl hydrolases family 32 protein\	[75,77]
VvAI2	GSVIVT0100127200	Vitvi02g00512	ACID BETA-FRUCTOFURANOSIDASE 3, VACUOLAR-RELATED	AT1G12240.1	ATBETAFRUCT4,VAC-INV	Glycosyl hydrolases family 32 protein\	[75,77]
